# Probing the orthogonality and robustness of the mammalian RNA-binding protein Musashi-1 in *Escherichia coli*

**DOI:** 10.1186/s13036-024-00448-x

**Published:** 2024-09-30

**Authors:** Roswitha Dolcemascolo, Raúl Ruiz, Sara Baldanta, Lucas Goiriz, María Heras-Hernández, Roser Montagud-Martínez, Guillermo Rodrigo

**Affiliations:** 1 Institute for Integrative Systems Biology (I2SysBio), CSIC – University of Valencia, Paterna, 46980 Spain; 2 Pure and Applied Mathematics University Research Institute (IUMPA), Polytechnic University of Valencia, Valencia, 46022 Spain

**Keywords:** Post-transcriptional regulation, RNA recognition motif, Synthetic biology, Systems biology

## Abstract

RNA recognition motifs (RRMs) are widespread RNA-binding protein domains in eukaryotes, which represent promising synthetic biology tools due to their compact structure and efficient activity. Yet, their use in prokaryotes is limited and their functionality poorly characterized. Recently, we repurposed a mammalian Musashi protein containing two RRMs as a translation regulator in *Escherichia coli*. Here, employing high-throughput RNA sequencing, we explored the impact of Musashi expression on the transcriptomic and translatomic profiles of *E. coli*, revealing certain metabolic interference, induction of post-transcriptional regulatory processes, and spurious protein-RNA interactions. Engineered Musashi protein mutants displayed compromised regulatory activity, emphasizing the importance of both RRMs for specific and sensitive RNA binding. We found that a mutation known to impede allosteric regulation led to similar translation control activity. Evolutionary experiments disclosed a loss of function of the synthetic circuit in about 40 generations, with the gene coding for the Musashi protein showing a stability comparable to other heterologous genes. Overall, this work expands our understanding of RRMs for post-transcriptional regulation in prokaryotes and highlight their potential for biotechnological and biomedical applications.

**Supplementary Information:**

The online version contains supplementary material available at 10.1186/s13036-024-00448-x.

## Introduction

Synthetic biology has progressed over the last two decades due to our increasing ability to engineer heterologous gene expression control systems [1]. By borrowing genetic elements from different organisms, the construction of bespoke genetic circuits to reprogram living cells and engineer novel biological functions has been possible, thereby unlocking a plethora of opportunities for applications ranging from medicine and agriculture to environmental remediation and industrial biomanufacturing [2–4]. However, recombinant protein expression usually faces the challenges of genetic compatibility, toxicity and burden, and correct folding and solubility [5]. This is especially relevant when the donor and recipient organisms are set apart phylogenetically.

Regulatory proteins able to target nucleic acids (DNA or RNA) are particularly appealing in synthetic biology to be borrowed because they boost the engineering of complex circuitries with sophisticated functionalities [1]. Most of the work has been done in the transcriptional layer, but the implementation of efficient and manageable post-transcriptional regulations with proteins acting as translation factors has been accomplished in recent years in bacteria [6–8]. Some CRISPR systems have even been repurposed to regulate gene expression at the post-transcriptional level [9–11]. Indeed, the combination of regulations at different points of the genetic information flow appears to be instrumental to achieve high signal integration and then obtain more compact circuits to deal with the trade-off between functional complexity and cellular burden.

In eukaryotes, of note, a large number of RNA-binding protein families exist [12], with potential for synthetic biology developments. One type of relevant proteins corresponds to those that contain RNA recognition motifs (RRMs), which are small globular domains of ~90 amino acids folding into four antiparallel β-strands and two α-helices with the ability to recognize single-stranded RNA with sufficient affinity and specificity [13]. Recently, we engineered a genetic circuit in *Escherichia coli* in which a Musashi protein from *Mus musculus* was used to repress the expression of a superfolder green fluorescent protein (sfGFP) at the post-transcriptional level thanks to a strong protein-RNA interaction [8]. Musashi proteins contain RRMs in their N-terminal regions (*viz*., a main domain leading RNA recognition, RRM1, and a subsidiary domain adding affinity, RRM2) and possess a strong affinity (at the nanomolar scale) for RNA sequences bearing the consensus pattern RU_*n*_AGU (*n* = 1-3) [14].

In that case, to enhance the compatibility, the sequence encoding the regulatory protein was codon optimized; to reduce the burden, the protein was expressed from a tightly regulated promoter induced with lactose or isopropyl β-D-1-thiogalactopyranoside (IPTG); and to increase the solubility, the sequence was truncated to remove the disordered C-terminal tail [8]. However, it is doubtful to what extent the expression of a Musashi protein affects the gene expression profile and intracellular functionalities of *E. coli* (*e.g*., metabolic, regulatory, or structural). Moreover, it is unknown whether the Musashi expression and regulatory action are robust enough in this prokaryotic context in order to be exploited as a biotechnological tool, such as to control bioproduction pathways given the responsiveness to fatty acids [15].

The impact of synthetic constructs on host cell physiology and *vice versa* is a matter that has been studied. For instance, in the case of systems expressed from plasmids, copy number is known to impact metabolic burden and circuit functionality, sometimes in opposite direction, and only minor effects on the biosynthesis machinery are typically noticed when dealing with orthogonal regulators [16]. Notably, the fusion of certain domains to the heterologous protein of interest can serve to mitigate a potential toxicity while maintaining the regulatory functionality, as demonstrated with the reengineering of the β-estradiol-responsive factor in *Saccharomyces cerevisiae* [17]. In addition, a large-scale characterization of heterologous transcription factors (TetR homologues) in *E. coli* revealed an ability by some of them to recognize non-cognate promoter sequences due to plastic DNA-binding domains [18], which stresses the necessity for controlling off-targets in the host genome. Nevertheless, a detailed study about the impact of heterologous RNA-binding proteins in bacteria is lacking. This seems relevant because bacteria possess a limited number of this type of proteins in their regulatory layer of gene-specific scope [19], in stark contrast with higher eukaryotes.

In this work, we combine genetic engineering, high-throughput RNA sequencing (RNA-seq), and experimental evolution to characterize the orthogonality and robustness of a mammalian Musashi protein in bacteria. We aim at deciphering if Musashi regulates endogenous genes in *E. coli* as a result of spurious protein-RNA interactions, if it creates a burden upon expression that ends in changes in the genetic profile of the cell and misregulation of certain biological processes, if the dynamic regulatory range can be modulated with mutations known to affect its RNA binding ability *in vitro*, or if it is evolutionarily stable. Collectively, our results shed light on the use of Musashi proteins in synthetic biology.

## Results

### Measuring the impact of expressing a Musashi protein on the transcriptomic and translatomic profiles of *E. coli*

**Fig. 1:**
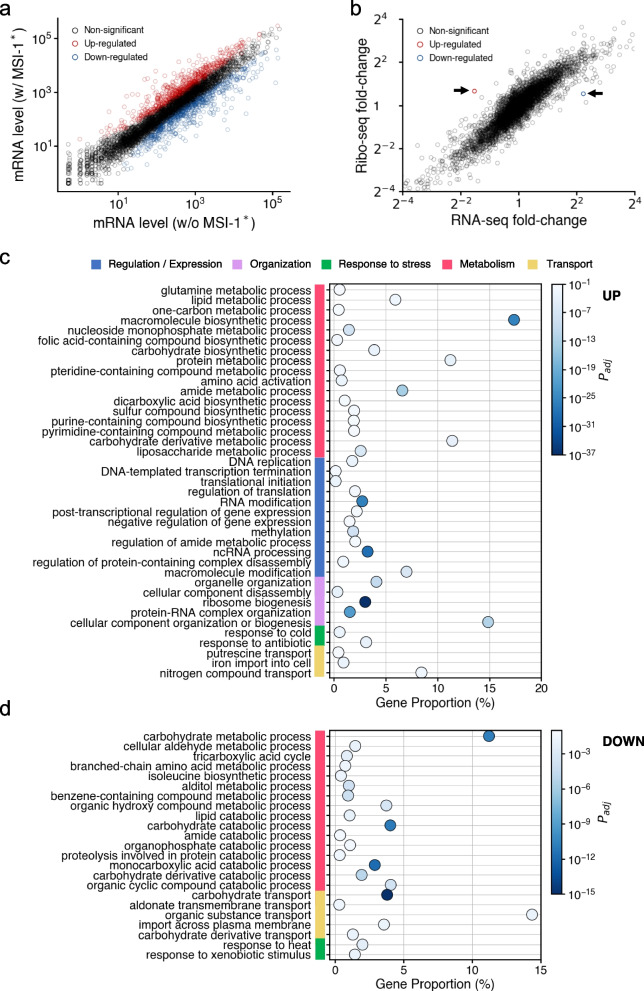
Differential gene and protein expression upon MSI-1* expression by RNA-seq and Ribo-seq. **a** Scatter plot of the expression of all host genes with and without MSI-1* (average of two replicates from RNA-seq). Transcriptionally up-regulated genes marked in red and transcriptionally down-regulated genes in blue. **b** Scatter plot of the fold change in RNA and protein expression (from RNA-seq and Ribo-seq, respectively) of all host genes upon MSI-1* expression (average of two replicates). Gene with up-regulated translation marked in red and gene with down-regulated translation in blue. **c** Functional analysis for the transcriptionally up-regulated genes. A relationship between gene proportion and adjusted *P* value is presented. **d** Functional analysis for the transcriptionally down-regulated genes

The gene expression profile of *E. coli* cells upon Musashi protein expression was analyzed. A truncated version of the Musashi-1 protein from *M. musculus* (termed MSI-1*, containing two RRMs) was expressed from a synthetic PL-based promoter (termed PLlac) in a high copy number plasmid. For that, we used *E. coli* cells with a knockout of the *LacI* gene, thereby ensuring a constitutive expression of MSI-1*. We employed RNA-seq to quantify the whole transcriptome of the cell with and without Musashi protein (each condition was measured in duplicate). We found 643 genes whose expression was significantly up-regulated in cells expressing MSI-1* (statistical significance assessed by a Wald test with Benjamini-Hochberg’s correction, *P* value < 0.05, and fold change > 1.5), whereas 616 genes whose expression was significantly down-regulated (Fig. 1a; see also Fig. S1). Among the up-regulated genes with larger fold change, we found genes coding for ribosomal proteins (*e.g.*, *rpsR* or *rlmF*), genes related to sulfate metabolism and transport (*e.g.*, *cysJ* or *cysP*), and genes coding for efflux pumps (*e.g*., *mdfA*). This suggested that *E. coli* deployed a general response following the stress imposed by MSI-1* expression [20]. In contrast, among the down-regulated genes with larger fold change, we found genes involved in the tryptophan synthesis and transport (*e.g*., *tnaB*), genes related to fructose utilization (*e.g*., *fruA*), and genes involved in the β-oxidation pathway by which energy is released from fatty acids (*e.g.*, *fadE* or *fadB*) [21].

To inspect in a comprehensive way the biological meaning of these alterations, we assessed the enrichment of certain processes within the list of differentially expressed genes upon MSI-1* expression taking advantage of gene ontology (GO) resources [22]. In the case of up-regulation, we found regulation of translation, response to antibiotic, as well as a series of metabolic processes (Fig. 1c). Synthetic gene circuits tend to stimulate the translation machinery of the host cell, in agreement with previous work [16]. Among the down-regulated genes, we mainly found catabolic processes of lipids and carbohydrates, transport of carbohydrates, and response to heat (*e.g*., *dnaK* was substantially down-regulated; Fig. 1d). Because DnaK is a negative modulator of heat-shock protein expression, such proteins were up-regulated in response to MSI-1* as a general mechanism against heterologous protein expression [23].

In addition, we employed ribosome profiling (Ribo-seq) [24] to quantify the change of the whole translatome upon expressing MSI-1*. We identified only two translationally regulated genes by normalizing the Ribo-seq counts by the RNA-seq counts (Fig. 1b). That is, *lyxK*, encoding a sugar kinase with up-regulated translation, and *plsX*, encoding a phosphate acyltransferase with down-regulated translation. The rest of the changes in protein expression were attributed to changes in RNA abundance (*i.e*., transcriptional effects; Fig. 1b). We searched the consensus pattern RU_*n*_AGU (*n* = 1-3) in duplicate, potentially recognized by the whole protein, along the host transcriptome, finding 43 messenger RNAs (mRNAs) harboring the intended sequence (Fig. S2). For such a task, we used the *E. coli* K-12 transcriptome from EcoCyc as a reference encompassing all known transcriptional units [25]. Among those identified mRNAs, we did not find *lyxK* or *plsX*. Isolated single instances of the consensus pattern were found within the transcripts involving *lyxK* and *plsX*, but within the coding region of other genes (Fig. S3). This suggested that those changes were due to indirect influences caused by the cost of expressing the heterologous protein. These high-throughput results help to describe in quantitative terms the impact of MSI-1* on the intracellular pathways of *E. coli*.

### Measuring the impact of mutations in a Musashi protein on the dynamic regulatory range of an engineered circuit

**Fig. 2:**
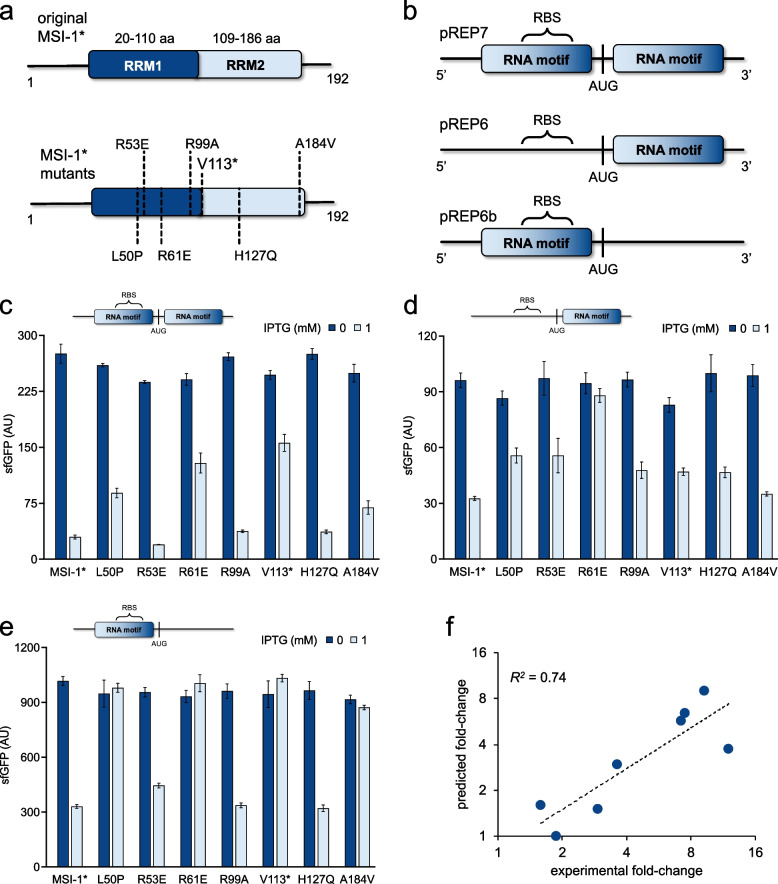
Impact of mutations in MSI-1* on the regulatory activity. **a** Scheme of the MSI-1* gene showing the different point mutations introduced. **b** Scheme of the different 5’ UTRs considered (containing RNA motifs). **c**-**e** Dynamic ranges of the responses upon induction with IPTG (1 mM) of the different genetic systems. pRM1+ and pREP7 in **c**), pRM1+ and pREP6 in **d**), and pRM1+ and pREP6b in **e**). Error bars correspond to standard deviations (three replicates). AU, arbitrary units. **f** Relationship between the predicted and experimental fold change for pRM1+ and pREP7. The predicted fold change for pRM1+ and pREP7 is the product of the experimental fold changes for pRM1+ and pREP6 and pRM1+ and pREP6b

We engineered a series of point mutations in the MSI-1* gene to study their impact on regulatory function in *E. coli* as a result of an altered RNA-binding capability (Fig. 2a). In particular, four mutations affected the RRM1 of the protein (L50P, R53E, R61E, and R99A), two affected the RRM2 (H127Q and A184V), and another one introduced a premature stop codon after the RRM1 (V113*). Most of these mutations were selected following previous work showing that they compromise RNA binding [15, 26, 27]. To perform the characterizations, we now used *E. coli* cells over-expressing LacI, thereby offering external control with IPTG as the PLlac promoter, which controls MSI-1* expression, is repressed by LacI. The high copy number plasmid harboring the MSI-1* gene was co-transformed with a low copy number plasmid from which to constitutively express a superfolder green fluorescent protein (sfGFP). This way, we forced a suitable ratio between the regulatory (protein) and regulated (RNA) molecules to ensure gene regulation. Two RNA motifs recognized by MSI-1* were introduced in the leader region of the *sfGFP* transcript, one in the ribosome binding site (RBS) and another after the start codon (Fig. 2b), to regulate its expression at the translation level.

The original protein showed 89.2% repression, and all mutants tested here, except the R53E one, displayed lower regulatory activity (Fig. 2c). Regarding the mutations affecting the RRM1, our results show that while the R53E and R99A mutation had minor effect on the performance (91.7% and 86.2% repression, respectively), both the L50P and R61E mutations reduced substantially the regulatory activity of MSI-1* (65.8% and 46.5% repression, respectively). According to previous structural simulations and *in vitro* assays, these four residues have been shown to establish contacts with small molecules, leading to allosteric regulation [15, 26]. Our data suggest that R61 and R99 also establish contacts with RNA, as well as they highlight the R53E mutant as a suitable regulator less sensitive to fatty acids [15] for further synthetic biology developments. For some mutations, we confirmed that they do not alter the expression level through a translational fusion of a red fluorescent protein (Fig. S4).

Regarding the mutations affecting the RRM2, our results show that both the H127Q and A184V mutations reduced moderately the performance (86.6% and 72.2% repression, respectively). Of note, Q127 is the native residue in the MSI-1 from *Homo sapiens*. Moreover, the A184V mutation has been shown to impede RNA binding in human cells, leading to deregulation of the target transcripts [27]. Notwithstanding, the use of a different sequence in the target mRNA, the lack of the C-terminal tail in the regulatory protein, and the prokaryotic context preclude establishing comparisons. Finally, the V113* mutation led to a 36.9% repression, suggesting that only one RRM is not enough to implement a post-transcriptional regulation with high dynamic range *in vivo*. This provides a rationale on why RRMs are typically found as repeated domains (from two to six) within a protein [13].

We also characterized the performance of the different mutants considering additional reporter systems in which only one RNA motif was present in the leader region of the *sfGFP* transcript (Fig. S5 shows the corresponding sequences and secondary structures). Figure 2d shows the regulatory activity when the motif is after the start codon, while Fig. 2e shows the activity when it is in the RBS. Assuming a scenario of independent binding, the fold change of the response with a two-motif target was well predicted by the product of the fold changes of the responses with one-motif targets (Fig. 2f; see also Fig. S6 where we depict the mathematical modeling of the response). Moreover, the reporter system lacking a hairpin after the start codon exhibited much higher expression levels [28]. Together, these results served to evaluate the mutational robustness of the heterologous RNA-binding protein.

### Measuring the evolutionary stability of an engineered circuit involving a Musashi protein in* E. coli*

To evaluate the evolutionary stability of the system (*i.e*., its ability to behave as designed with time), we set up an experiment of serial dilutions by carrying in parallel 20 different bacterial populations during one week (using cells over-expressing LacI). In particular, 10 populations were evolved without IPTG (*i.e*., maintaining repressed the MSI-1* gene), while another 10 populations were exposed to IPTG (*i.e*., expressing MSI-1* in high amounts). We considered two different plasmids to express MSI-1*, one of high copy number and another of medium copy number. Every day, circuit functionality and stability were assessed by fluorometry and Sanger sequencing.

**Fig. 3:**
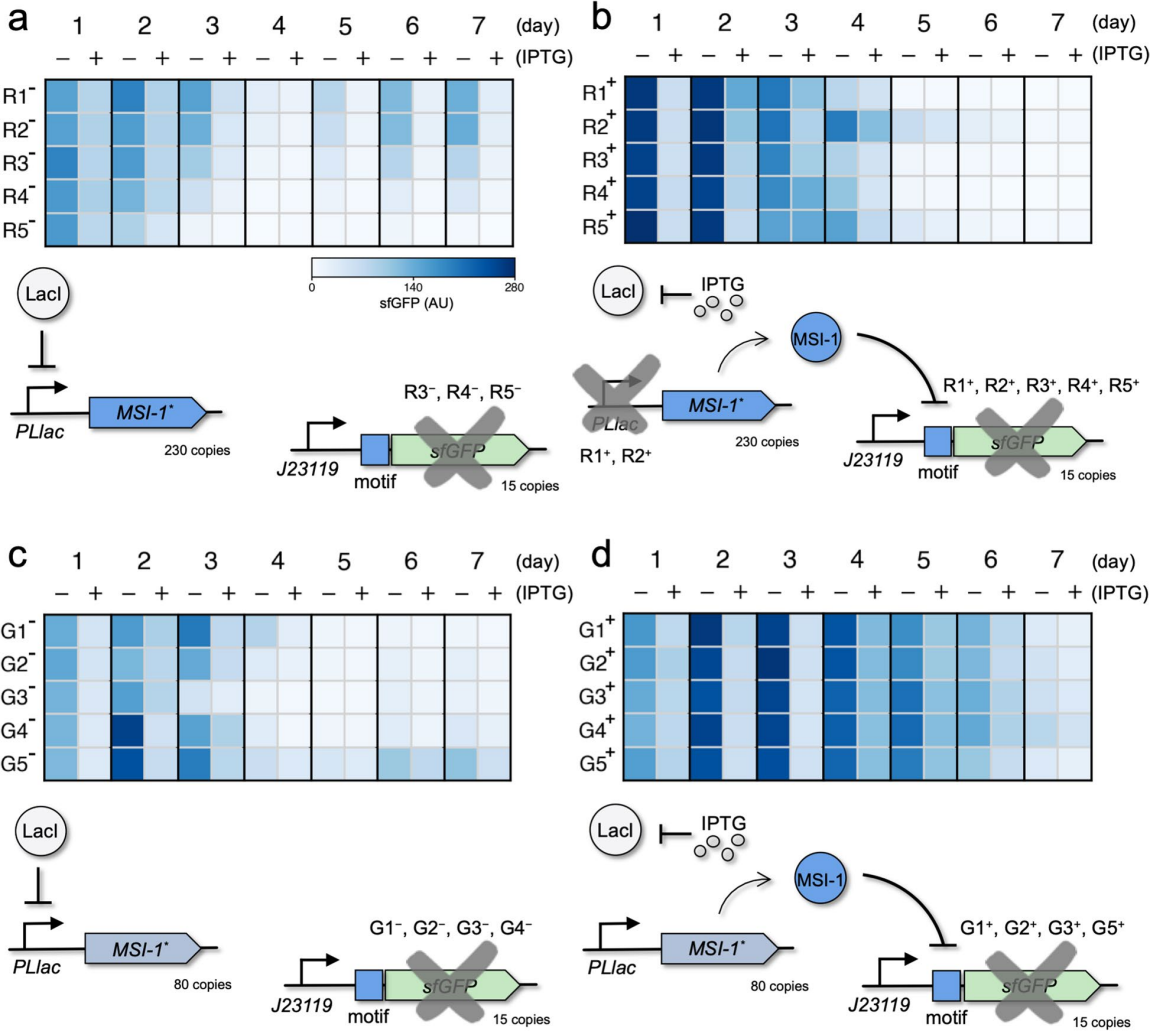
Evolutionary stability of an engineered circuit involving MSI-1*. **a** Heatmap of the daily dynamic ranges (average of three replicates) of the responses upon induction with IPTG (1 mM) of the different genetic systems evolved during 7 d (46.5 generations). Data for five lines of cells co-transformed with pRM1+ and pREP7 evolved in a medium without IPTG. On the bottom, scheme of the genetic circuit. Crosses indicate that the genetic element accumulated mutations with time. **b** Heatmap of the daily dynamic ranges of the responses upon induction with IPTG. Data for five lines of cells co-transformed with pRM1+ and pREP7 evolved in a medium with IPTG. **c** Heatmap of the daily dynamic ranges of the responses upon induction with IPTG. Data for five lines of cells co-transformed with pRM1 and pREP7 evolved in a medium without IPTG. **d** Heatmap of the daily dynamic ranges of the responses upon induction with IPTG. Data for five lines of cells co-transformed with pRM1 and pREP7 evolved in a medium with IPTG. AU, arbitrary units

We found that in absence of IPTG, when sfGFP was expressed but not MSI-1*, the system accumulated mutations in the form of deletions in the *sfGFP* gene in most of the evolved lines, thereby abolishing the fluorescence signal (Fig. 3a,c; see also Fig. S7). As expected, this occurred irrespective of the plasmid copy number of the regulator. In these conditions, and considering 6.64 generations per day from a 1% bottleneck, the evolutionary stability of the *sfGFP* gene was assessed in about 20 generations as fluorescence declined in about 3 days (note that the plasmid copy number of the reporter is low). No mutations were detected in the MSI-1* gene, and the dynamic regulatory range of the system (*i.e*., repression fold obtained by inducing with IPTG) was roughly preserved during such first generations.

In presence of IPTG during evolution, when MSI-1* was expressed and sfGFP repressed at the translation level, the results were different. When the regulator was expressed from a high copy number plasmid, the system accumulated mutations in the form of deletions in the PLlac promoter and in the *sfGFP* gene (Fig. 3b; see also Fig. S8). As previously shown, the two LacI operators within the promoter triggered a homologous recombination event that removed the -35 region [29], so MSI-1* was no longer expressed. In this case, the fluorescence signal was lost in about 26 generations. However, when the regulator was expressed from a medium copy number plasmid, the system only accumulated mutations in the *sfGFP* gene (Fig. 3d; see also Fig. S8). The PLlac promoter remained stable and no mutations were detected in the MSI-1* gene, which suggested a correct MS1-1* expression with time. Certainly, synthetic circuit stability is usually increased by reducing the plasmid copy number [30]. In this latter case, a proper dynamic range of fluorescence held for up to 40 generations. In the light of these results, MSI-1* expression arguably created less burden to the cell than sfGFP expression. Hence, the MSI-1* gene seems sufficiently stable in *E. coli* to be used in synthetic biology applications.

## Discussion

Our analysis followed previous work that explored the utilization of the mammalian RNA-binding protein MSI-1 as a translation regulatory factor in *E. coli* [8]. Here, we employed RNA-seq and Ribo-seq to investigate the impact of MSI-1* expression on the host cell in terms of differential gene or protein expression and associated functional enrichment. Moreover, we performed evolutionary experiments and explored the effect on the regulation of different point mutations in MSI-1* and different 5’ UTR configurations to characterize the circuit robustness.

Our study unveiled that MSI-1* presumably interferes with the β-oxidation pathway and induces post-transcriptional regulatory processes, on top of leading to a roughly generalist stress response in the bacterium by perturbing its metabolic state. A competition for resources at the translation level (ribosomes) between the host systems and the synthetic circuit, driven by the expression of heterologous proteins, was expected, as already pointed out [31] and illustrated by the fact that some GO terms associated with translation were significantly perturbed. We did not find evidence that MSI-1* regulated post-transcriptionally in a direct way any host gene in the bacterium, as only two genes showed altered translation rate but without harboring RNA motifs. These results align with a known general response of *E. coli* to the stress generated by the expression of recombinant proteins, which involves the adjustment of the protein production system, the stimulation of heat-shock response proteins, the increase of the ppGpp concentration, and the induction of plasmid instability, with overlapping regulatory networks in charge of deploying the appropriate response [20]. Importantly, our study illustrates how global-scale measurements (*i.e*., RNA-seq and Ribo-seq) are useful to debug and characterize the operability regime of a synthetic regulatory circuit and its interaction with the host cell [16, 32]. This experimental strategy could be complemented with computational circuit-host interaction models to predict the impact of design parameters on burden and functionality [33]. This is intended to engineer low-power circuit variants to minimize their influence on the host cell.

In addition, we showed that most of the engineered MSI-1* mutants had compromised regulatory behavior (in comparison to the original protein). Both RRMs within MSI-1* were required for a ~10-fold repression of translation (see the performance of the V113* mutant), in tune with prior biochemical and structural findings that showed that RRM1 determines RNA binding specificity whereas RRM2 adds affinity [34]. It is important to highlight that the R53 residue was shown key to respond to fatty acids [15], but the R53E mutant preserved the regulatory activity by its RNA binding ability. In terms of evolutionary stability, we noticed that the engineered circuit maintained a proper dynamic range of fluorescence for up to 40 generations when MSI-1* was expressed from a medium copy number plasmid. The strong binding of the protein to the target RNA (*viz*., at the nM level) [8, 14] allowed this reduction in copy number while maintaining the dynamic range of the response. Our results also showed that the MSI-1* gene is not less stable in *E. coli* than a typical *GFP* gene, prompting further biotechnological developments with MSI-1*.

Synthetic biology has broadened the bioproduction of chemicals with biomedical or industrial application in bacteria. A key challenge in microbial factory design is balancing the distribution of metabolic fluxes to prevent the accumulation of undesirable or harmful metabolites that can impair cell growth [35]. To address this challenge, precise control over gene expression becomes imperative. In this regard, MSI-1* could be used for finely tuning enzyme production without altering transcription levels. This could boost the implementation of combinatorial regulation (*i.e.*, multiplexing the action of transcription and translation factors) and the expression divergence of genes within the same operon [8]. In addition, considering the importance of Musashi proteins in animals, as their malfunction can lead to neurodegeneration and cancer [36], we anticipate that our synthetic system could be exploited to characterize naturally-occurring mutations in both the protein and the target RNA, with potential application in biomedicine. Indeed, a cellular system in which the elements at play are genetically encoded allows avoiding protein purification each time and allows having a fluorescent reporter to perform large-scale screenings.

In sum, this study contributes to enlarge our understanding about the use of RNA-binding proteins in bacteria to implement post-transcriptional regulation of protein expression. Despite these elements have attracted less attention than small RNAs to perform synthetic biology developments [37], they are equally valid and may exhibit higher specificity for their targets. As more transcription and translation factors are fully characterized, more and more complex gene circuits will be engineered to end with functional bacteria meeting a variety of demands. In the particular case of MSI-1*, given its regulatory activity and responsiveness to fatty acids, we envision its application to engineer control systems of metabolic pathways in bacteria aimed to produce high-value biochemicals for the energy and pharmaceutical industries.

## Methods

### Strains, plasmids, and reagents

*E*. *coli* MG1655-Z1 cells (*LacI*^+^, *TetR*^+^) were used to express our genetic circuit for functional characterization. *E. coli* JS006 cells (MG1655, *LacI*^−^, *AraC*^−^) were used to express MSI-1* and characterize its impact on the host by RNA-seq and Ribo-seq. *E*. *coli* Dh5α cells were used for cloning purposes following standard procedures.

The circuit was composed of two different plasmids, one expressing MSI-1* and another expressing sfGFP. In particular, pRM1 (KanR, pSC101-E93G ori; leading to ~80 copies/cell) or pRM1+ (KanR, pSC101-E93R ori; leading to ~230 copies/cell) were used to express MSI-1* [38], and pREP7 (CamR, p15A ori; leading to ~15 copies/cell) was used to express sfGFP. Figure S9 shows the maps of the main plasmids used in this work. Additional plasmids to express MSI-1* mutants were constructed by PCR; the resulting plasmids were named pRM1+_stop, pRM1+_53, pRM1+_61, pRM1+_99, pRM1+_127, and pRM1+_184 (Table S1 shows the oligonucleotide sequences used to perform the site-directed mutagenesis reactions). Moreover, another two reporter plasmids were considered, *viz*., pREP6 and pREP6b, with different RNA motif sequences to be recognized by MSI-1* (the latter constructed in this work). Finally, three more plasmids were designed and cloned (pRM1+_mScarlet, pRM1+_61_mScarlet, and pRM1+_184_mScarlet) containing a translational fusion between MSI-1* and mScarlet to demonstrate equal expression levels after mutation. Plasmids were amplified by PCR to insert the *mScarlet* gene with a glycine-serine linker (Table S2).

Luria-Bertani (LB) medium was used for the overnight cultures and M9 minimal medium (1X M9 minimal salts, 2 mM MgSO_4_, 0.1 mM CaCl_2_, 0.05% thiamine, 0.05% casamino acids, and 0.4% glucose) for the characterization cultures. Kanamycin and chloramphenicol were used at a concentration of 50 μg/mL and 34 μg/mL, respectively. IPTG was used as the inducer of the system (controlling the expression of MSI-1* in *E. coli* MG1655-Z1) at a concentration of 1 mM. Compounds provided by Merck.

### Bulk fluorometry

*E*. *coli* MG1655-Z1 cell cultures (2 mL) inoculated from single colonies (in triplicate) were grown overnight in LB medium at 37 ºC and 200 rpm. Cultures were then diluted 1:100 in fresh M9 medium (200 μL) with and without IPTG. The microplate (96 wells, black, clear bottom; Corning) was incubated at 37 ºC with shaking (1300 rpm, 2 mm orbit) to reach an OD_600_ around 0.6. The microplate was assayed in a Varioskan Lux fluorometer (Thermo Fisher Scientific) to measure absorbance (600 nm) and green fluorescence (excitation: 485 nm, emission: 535 nm). Background values of absorbance and fluorescence, corresponding to M9 medium, were subtracted to correct the signals. The normalized fluorescence was calculated as the ratio between the corrected fluorescence and absorbance. The normalized fluorescence of non-transformed cells was subtracted to obtain a final estimate of expression.

### RNA-seq and Ribo-seq

*E. coli* JS006 cells transformed with pRM1+ or with a void plasmid (in duplicate) were grown in LB medium at 37 ºC to reach an OD_600_ around 0.4. Cells were harvested from 200 mL of culture by rapid filtration through a Kontes 90 mm filtration apparatus with 0.45 μm nitrocellulose filters (Whatman). Cells were scraped from the filter in two aliquots (90% for Ribo-seq and 10% for RNA-seq) before being immediately frozen in liquid nitrogen. Total RNA was extracted from RNA-seq aliquots in TRIzol and ribosomal RNA (rRNA) was removed, then mRNAs were fractionated and converted into Illumina compatible cDNA libraries. Ribo-seq aliquots were lysed in 600 μL ice-cold polysome lysis buffer (Tris-based) by bead beating in a FastPrep-24 with CoolPrep Adapter (3 rounds at 6 m/s for 30 s in 2 mL cryovials containing 0.1 mm silica beads). Lysates were clarified by centrifugation at 10,000 g for 5 min at 4 ºC. Ribosomes were subsequently pelleted from lysates by ultracentrifugation at 370,000 g for 1 h at 4 ºC and resuspended in polysome digestion buffer (Tris-based). Samples were then digested with 750 U MNase for 1 h at 25 ºC and the reaction was stopped by adding 6 mM EGTA. RNA was purified and ribosome-protected mRNA fragments ranging 20-40 nt in length were selected for size using 15% urea PAGE gels. rRNA was depleted from samples using custom biotinylated rRNA depletion oligos for *E. coli*. The enriched fragments were then converted into cDNA libraries compatible with Illumina sequencing. RNA-seq and Ribo-seq libraries (for mRNAs) were generated by EIRNA Bio. Both libraries were sequenced (paired-end 150 bp) on the Illumina’s Nova-seq 6000 platform to depths of 10 M and 30 M raw read pairs per sample, respectively (Dataset S1 contains the count data). Raw data deposited in the GEO database (refs. GSE275581 and GSE275582).

### Differential expression analysis

The BioConductor R package DESeq2 was used to perform the differential gene expression analysis [39]. The statistical significance was evaluated by means of a Wald test in pair-wise data (*i.e*., two conditions) with a correction for multiple testing using the Benjamini-Hochberg’s FDR. An adjusted *P* value of 0.05 was used as a cutoff value, also ensuring a minimal fold change of 1.5. Differences in translation efficiency (*i.e*., comparing Ribo-seq with RNA-seq data) were evaluated as previously proposed [40].

From the lists of *E. coli* genes whose expression (mRNA level) was significantly altered upon MSI-1* expression (up- and down-regulated), functional analyses were performed using the BioConductor R package clusterProfiler [41]. The statistical significance of the enriched GO terms, with respect to the complete *E. coli* K-12 genome, was evaluated by a Fisher’s exact test (2x2 contingency table) with a correction for multiple testing using the Benjamini-Hochberg’s FDR. An adjusted *P* value of 0.05 was used as a cutoff value. The lists of enriched GO terms were then processed using REVIGO [42] to reduce redundancy and cluster similar terms.

### Experimental evolution

*E*. *coli* MG1655-Z1 cell cultures (5 mL) were grown overnight in LB medium at 37 ºC and 200 rpm. Propagation of the lines during 7 d was carried out by imposing a daily 1% bottleneck (dilution 1:100), leading to 6.64 generations/d. Five lines of cells co-transformed with pRM1 and pREP7 evolved in a medium with 1 mM IPTG, five lines of cells co-transformed with pRM1 and pREP7 in a medium without IPTG, five lines of cells co-transformed with pRM1+ and pREP7 in a medium with 1 mM IPTG, and five lines of cells co-transformed with pRM1+ and pREP7 in a medium without IPTG. A daily fossil record of each line was generated by adding 20% glycerol to an equal volume of culture.

### Sequencing of the genetic circuit

To sequence the genetic cassette expressing MSI-1*, we used the following forward primer: GTGAGCCAGTGAGTTGATTGC. To sequence the genetic cassette expressing sfGFP, we used the following forward primer: CGCCCGGTAGTGATCTTATTTC. Sanger sequencing carried out by Eurofins Genomics.

## Supplementary Information

Additional file 1: Figs. S1-S9, Tables S1-S2. Supplementary figures and tables to provide additional information and results.

Additional file 2: Dataset S1. Count data of RNA-seq and Ribo-seq.

## Data Availability

Data provided in the supplementary information files. Raw data deposited in the GEO database (refs. GSE275581 and GSE275582).
