# Supplementary material for: Probing the orthogonality and robustness of the mammalian RNA-binding protein Musashi-1 in Escherichia coli
Source: J Biol Eng. 2024 Sep 30;18:52. doi: 10.1186/s13036-024-00448-x (PMC11443895; doi:10.1186/s13036-024-00448-x)
Supplement: Supplementary file 1 — Additional file 1: Figs. S1-S9, Tables S1-S2. Supplementary figures and tables to provide additional information and results. [file 13036_2024_448_MOESM1_ESM.pdf]

Supplementary Material

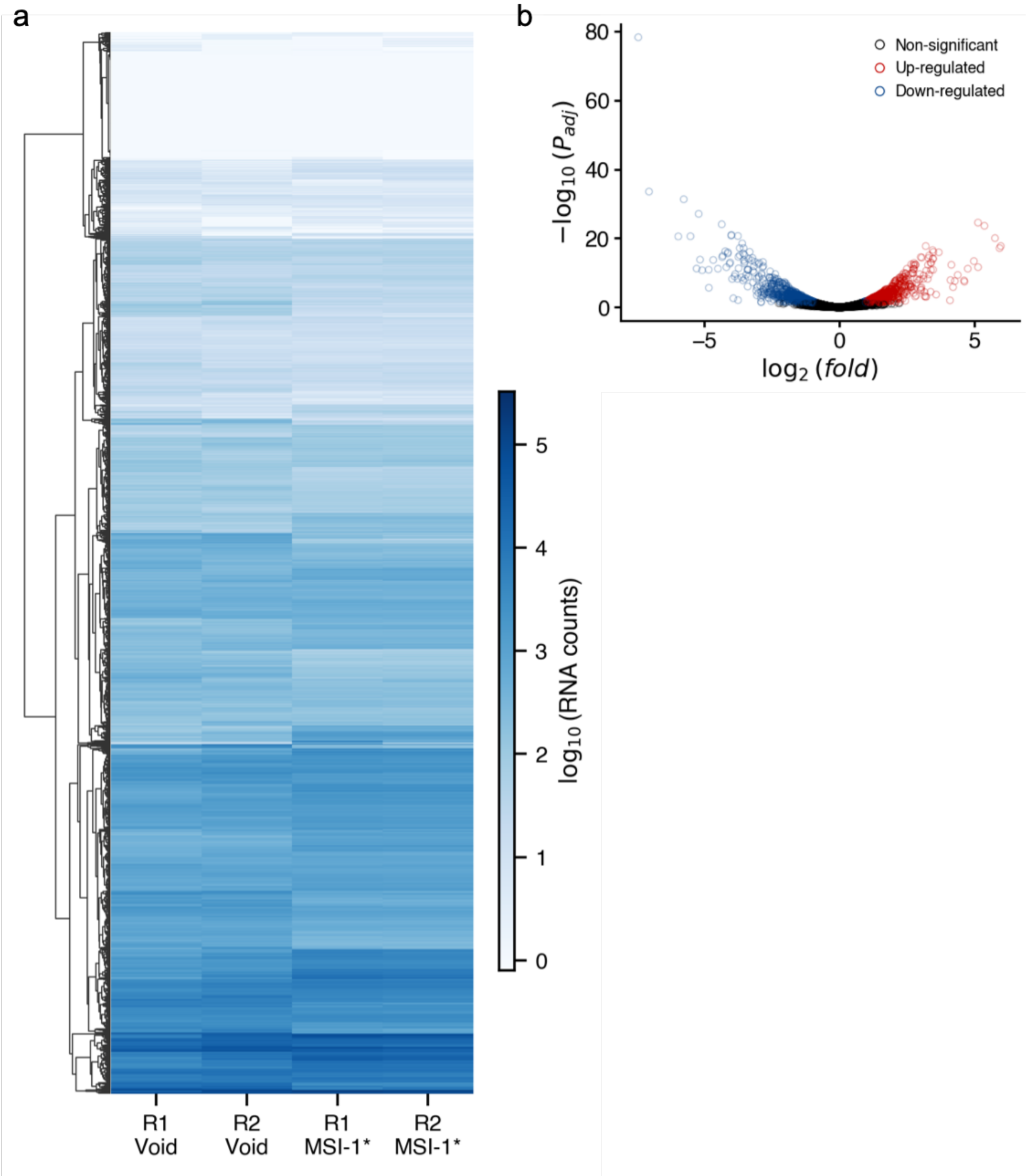

Fig. S1: a) Heatmap of gene expression in *E. coli* without and with MSI-1*. On the left, hierarchical clustering of the genes. b) Volcano plot showing the relationship between fold change in gene expression and adjusted *P* value (FDR).

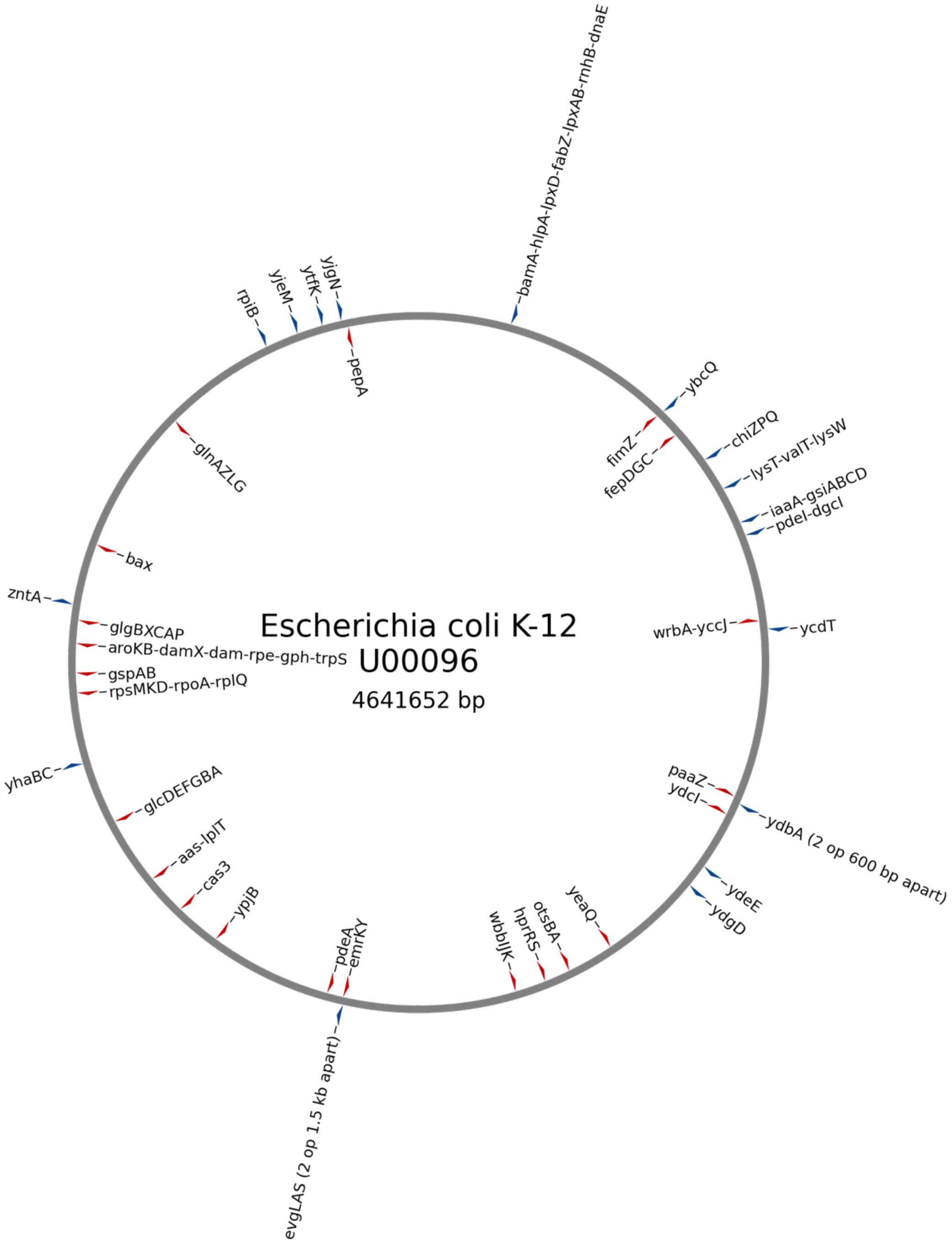

Fig. S2: Analysis of putative MSI-1* recognition motifs (for one RRM) in the *E. coli* transcriptome. The regular expression searched is $RU_nAGUN_kRU_nAGU$ (n = 1-3, k = 1-10). The transcriptional units in which the motifs were found are highlighted.

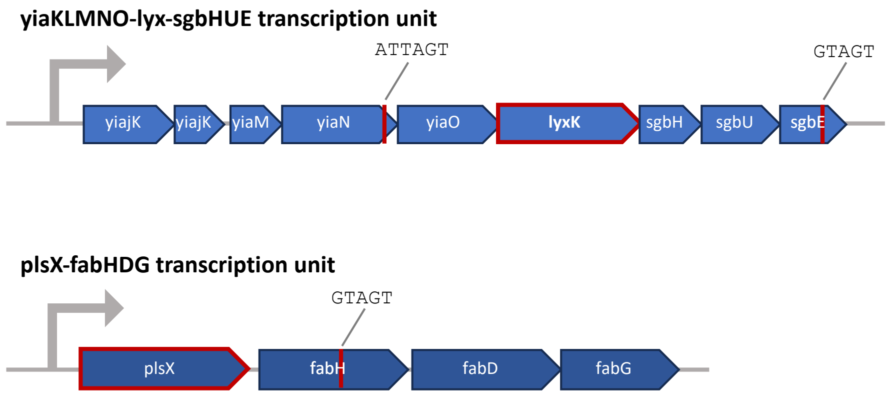

Fig. S3: Schematics of the transcriptional units of *lyxK* (top) and *plsX* (bottom). Putative MSI-1* recognition motifs (for one RRM) found in those regions are marked.

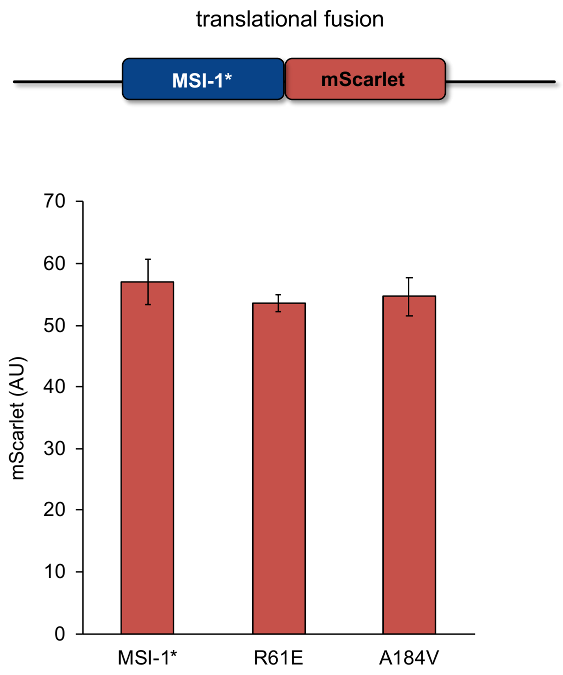

Fig. S4: Comparison of expression levels of different genetic systems (MSI-1* mutants). Error bars correspond to standard deviations (four replicates). No significant difference found by one-way ANOVA (P = 0.42).

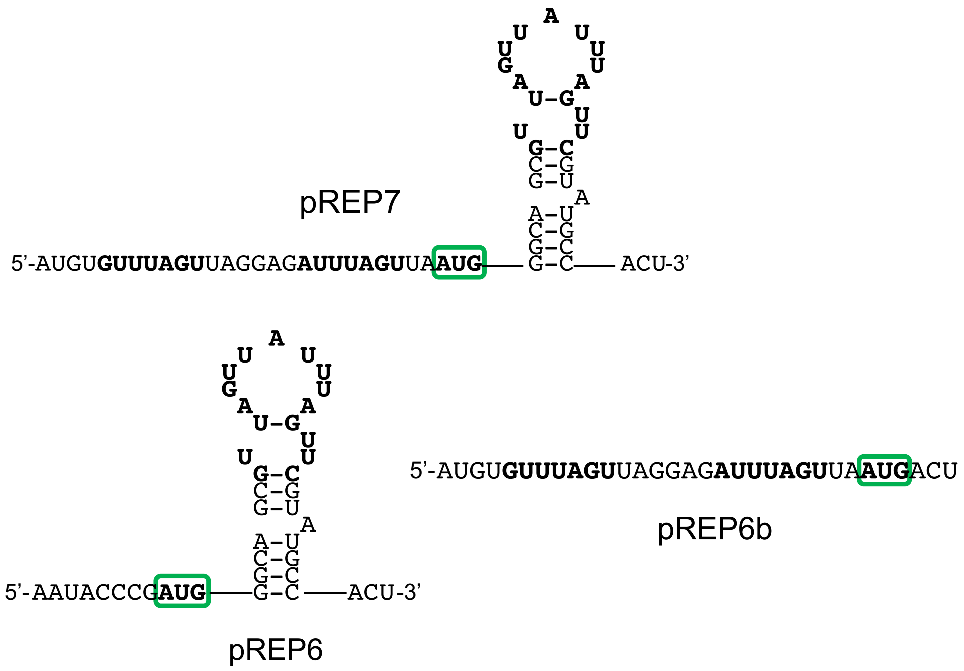

Fig. S5: RNA sequences and secondary structures of the different 5' UTRs used in the reporter systems (pREP7, pREP6, and pREP6b). The start codon is encircled in green.

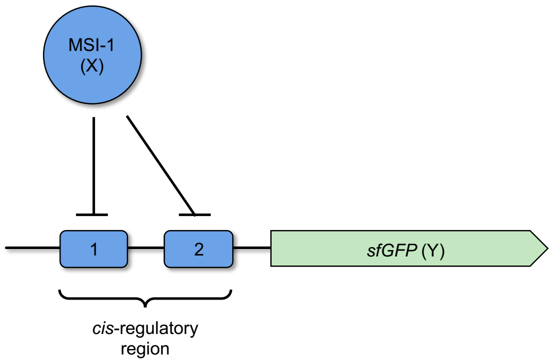

$$[Y] = \frac{1}{1 + \frac{[X]}{K_1} + \frac{[X]}{K_2} + \omega \frac{[X]^2}{K_1 K_2}}$$

$$fold = \frac{[Y]_{X=0}}{[Y]_X} = 1 + \frac{[X]}{K_1} + \frac{[X]}{K_2} + \omega \frac{[X]^2}{K_1 K_2}$$

$$\text{if } \omega = 1, \text{then } \; fold = \left(1 + \frac{[X]}{K_1}\right)\left(1 + \frac{[X]}{K_2}\right)$$

Fig. S6: Schematics of the regulatory action and mathematical modelling of expression. The regulator has a given affinity for each operator (K_1 and K_2). In the equation, ω models the interaction between regulators. If ω>1, cooperative binding. If ω<1, competitive binding (exclusive binding when ω=0). If ω=1, independent binding.

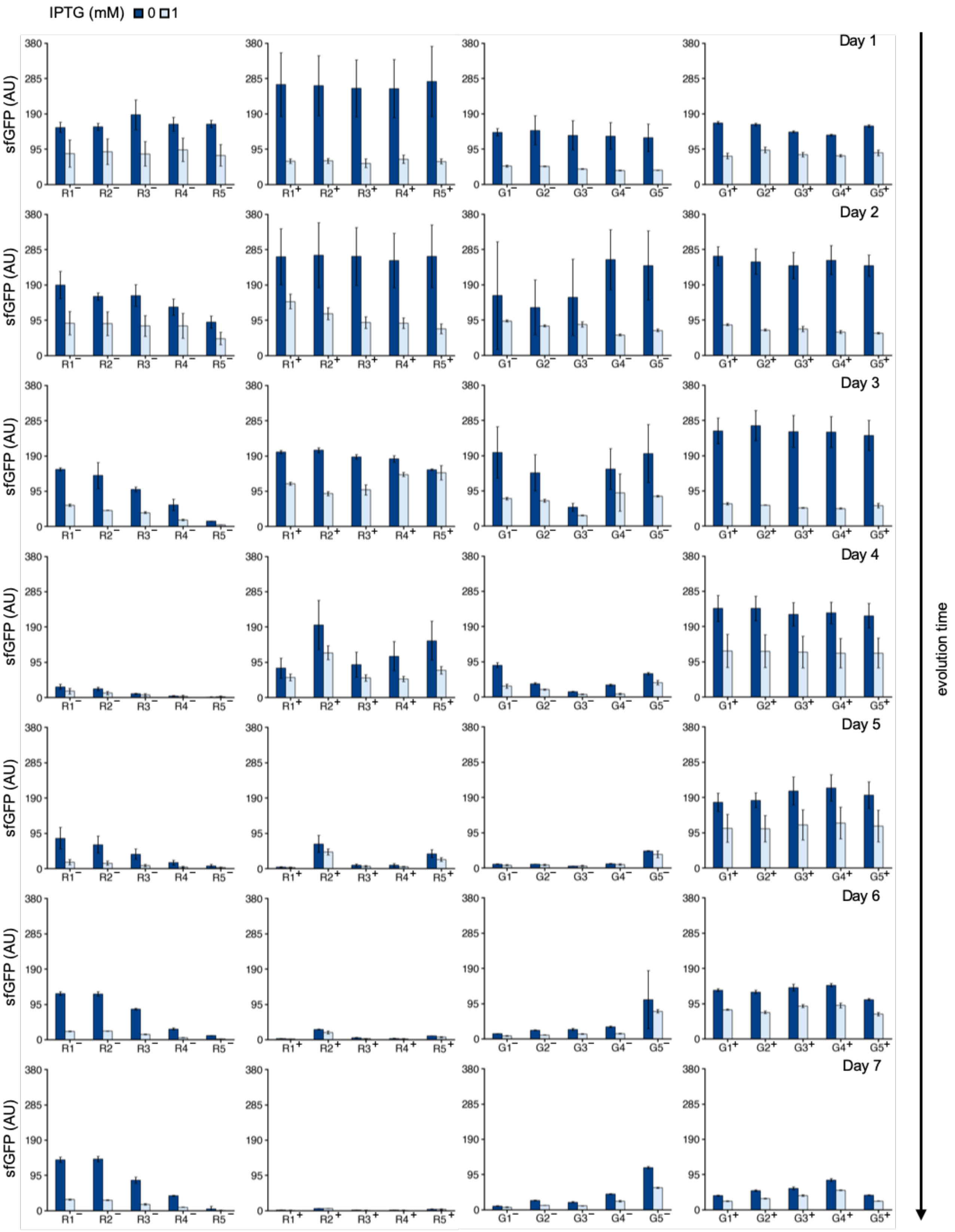

Fig. S7: Dynamic range of the responses upon induction with IPTG (1 mM) of the different genetic systems evolved during 7 d (46.5 generations).

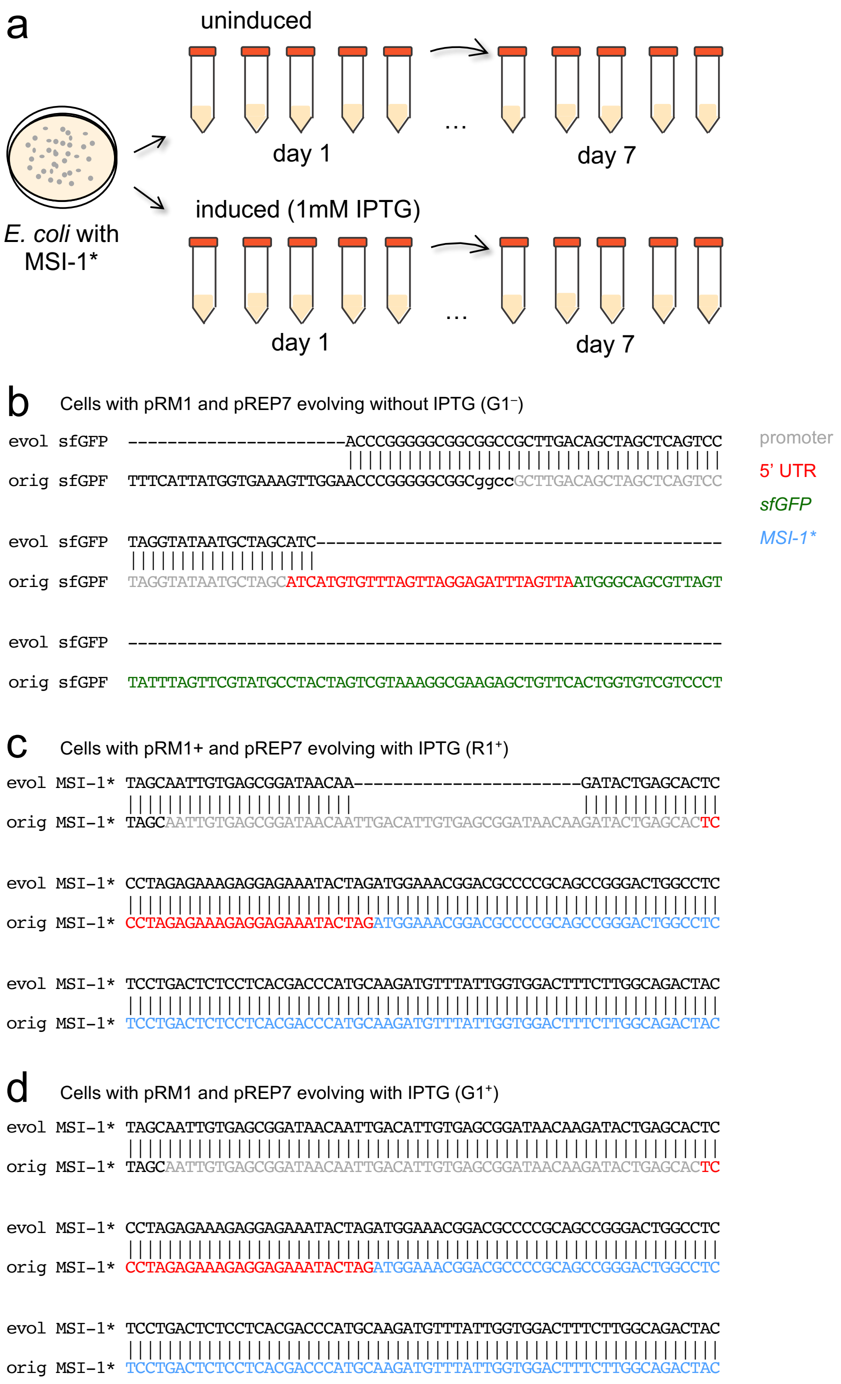

Fig. S8: a) Schematics of the experimental evolution process. b) Sequence alignment of the *sfGFP* cassette between the original and evolved cells. In this case, cells harbored pRM1 and pREP7 and evolved in a medium without IPTG (results for the representative line G1$^-$). c) Sequence alignment of the *MSI-1** cassette between the original and evolved cells. In this case, cells harbored pRM1+ and pREP7 and evolved in a medium with IPTG (results for the representative line R1$^+$). d) Sequence alignment of the *MSI-1** cassette between the original and evolved cells. In this case, cells harbored pRM1 and pREP7 and evolved in a medium with IPTG (results for the representative line G1$^+$).

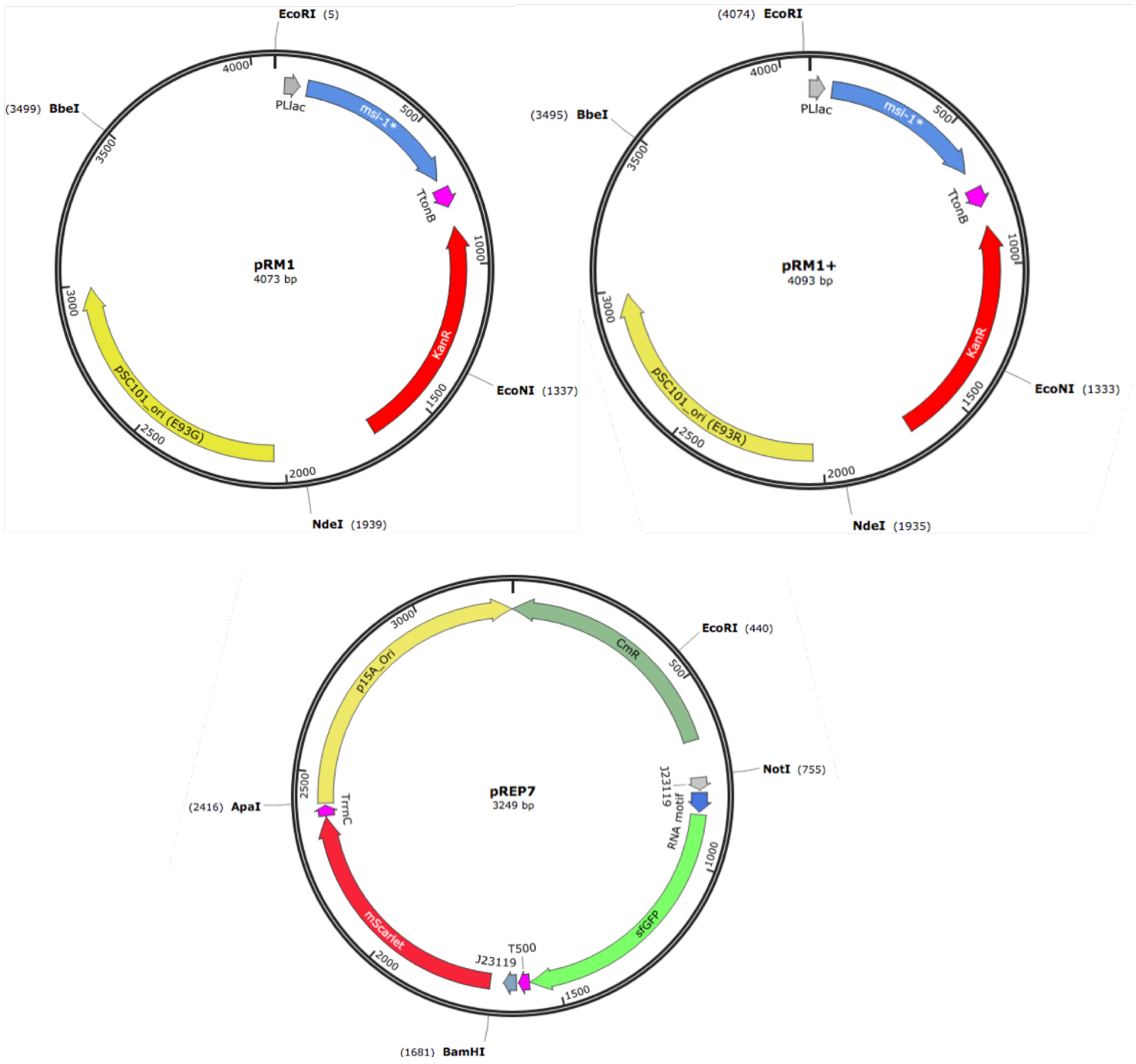

Fig. S9: Maps of the main plasmids (pRM1, pRM1+, and pREP7) used in this work. pRM1 or pRM1+ were used to express MSI-1* (the regulator of the circuit), controlled by IPTG (the input of the circuit). pREP7 was used to express sfGFP (the output of the circuit).

Table S1: Sequences of the oligonucleotides used to perform site-directed mutagenesis. The introduced mutations are in boldface in the forward oligo.

plasmid name	forward oligo (5' – 3')	reverse oligo (5' – 3')	backbone feature
pRM1+_R53E	GTCTTGTGATG**GAG**GATCCTTTAACC	CACAAGACACTCTTTCACTTCGCC	KanR, pSC101(E93R) ori
pRM1+_R61E	CCAAGCGTAGT**GAG**GGATTTGGC	CGCTTGGTTAAAGGATCGCGCATC	KanR, pSC101(E93R) ori
pRM1+_R99A	GTTCCCACGT**GC**CGCCCAAC	GGGAACGCCACTTTGGGGTC	KanR, pSC101(E93R) ori
pRM1+_V113*	CTCGTACCAAAAAGATTTTCTAGGAGGC	GGTACGAGTAACCATTTTAGGTTGG	KanR, pSC101(E93R) ori
pRM1+_H127Q	GAAGATGTAAAG**CAA**TACTTCGAACAG	CATCTTCTACCGTGGTATTTACG	KanR, pSC101(E93R) ori
pRM1+_A184V	GTAAGAAGG**T**GCAACCGAAAG	GTTTTACCAACTCACATTCTTCC	KanR, pSC101(E93R) ori

Table S2: Sequences of the oligonucleotides used to perform the translational fusion between MSI-1* and mScarlet.

product to amplify	forward oligo (5' – 3')	reverse oligo (5' – 3')	application
mScarlet gene	cggtACTAGTGTGAGCAAGGGCGA	GCGCTGCAGTTATCACTTGT	used to build pRM1+_mScarlet, pRM1+_61_mScarlet, and pRM1+_184_mScarlet
pRM1+, pRM1+_R61E, and pRM1+_A184V plasmids	atcctgcagAGGGTTAGTTAGTTAGATTAGC	tcacactagtaccggatccgccAGGAGACATGACTTCTTTCGGT*	used to build pRM1+_mScarlet, pRM1+_61_mScarlet, and pRM1+_184_mScarlet

*This oligo included the glycine-serine linker and served to remove the stop codon of the *MSI-1** gene.
